# Association Between Gut Microbiota and Autism Spectrum Disorder: A Systematic Review and Meta-Analysis

**DOI:** 10.3389/fpsyt.2019.00473

**Published:** 2019-07-17

**Authors:** Mingyu Xu, Xuefeng Xu, Jijun Li, Fei Li

**Affiliations:** ^1^Developmental and Behavioral Pediatric & Child Primary Care Department, Ministry of Education-Shanghai Key Laboratory of Children’s Environmental Health, Xinhua Hospital, Shanghai Jiao Tong University School of Medicine, Shanghai, China; ^2^Department of Pulmonology, Children’s Hospital, Zhejiang University School of Medicine, Hangzhou, China; ^3^Department of Integrative Medicine on Pediatrics, Shanghai Children’s Medical Center, Shanghai Jiao Tong University School of Medicine, Shanghai, China; ^4^Shanghai Institute of Pediatric Research, Xinhua Hospital, Shanghai Jiao Tong University School of Medicine, Shanghai, China

**Keywords:** autism spectrum disorder, children, GI problems, gut microbiota, microflora, meta-analysis

## Abstract

Autism spectrum disorder (ASD) is characterized by stereotyped behavior and deficits in communication and social interactions. Gastrointestinal (GI) dysfunction is an ASD-associated comorbidity, implying a potential role of the gut microbiota in ASD GI pathophysiology. Several recent studies found that autistic individuals harbor an altered bacterial gut microbiota. In some cases, remodeling the gut microbiota by antibiotic administration and microbiota transfer therapy reportedly alleviated the symptoms of ASD. However, there is little consensus on specific bacterial species that are similarly altered across individual studies. The aim of this study is to summarize previously published data and analyze the alteration of the relative abundance of bacterial genera in the gut microbiota in controls and individuals with ASD using meta-analysis. We analyzed nine studies, including 254 patients with ASD, and found that children with ASD had lower percentages of *Akkermansia*, *Bacteroides*, *Bifidobacterium*, and *Parabacteroides* and a higher percentage of *Faecalibacterium* in the total detected microflora compared to controls. In contrast, children with ASD had lower abundance of *Enterococcus*, *Escherichia coli*, *Bacteroides*, and *Bifidobacterium* and higher abundance of *Lactobacillus*. This meta-analysis suggests an association between ASD and alteration of microbiota composition and warrants additional prospective cohort studies to evaluate the association of bacterial changes with ASD symptoms, which would provide further evidence for the precise microbiological treatment of ASD.

## Introduction

Autism spectrum disorder (ASD) is a neurodevelopmental disorder characterized by stereotyped behavior and deficits in communication and social interactions. ASD is highly heterogeneous and its etiology is unclear. Previous studies have revealed several potential causes of this disease, such as genetic abnormalities, dysregulation of the immune system, inflammation, and environmental factors (1–5). Gastrointestinal (GI) problems, including constipation, abdominal pain, gaseousness, diarrhea, and flatulence, are common symptoms associated with ASD in a prevalence range from 23% to 70% (4, 6–8). Although there is no direct evidence that GI symptoms and ASD have a cause-effect relationship, studies have suggested that the gut has an important role in the etiology of ASD (9). Recently, interactions between the gut and the brain in ASD have received considerable attention (10–12). Over millennia, selected microbiota have become resident in the human GI tract, which is integrated with the immune system, metabolism, and nervous system (13, 14). These gut-adapted bacteria and their metabolites might have a critical role in the pathophysiology of ASD. Studies in rodents have indicated that the gut microbiota appears to influence the development of emotional behaviors and brain neurotransmitter systems, further suggesting the existence of a microbiota gut-brain axis (15–18). The gut microbiota has assumed its rightful position as a critical component of the brain-gut axis, highlighting its potential impact on behavior and mood at the level of the central nervous system (10). Furthermore, in some cases, remodeling the gut microbiota by antibiotic administration and microbiota transfer therapy reportedly alleviated the symptoms of ASD (19). The application of probiotics could influence microbiota composition and intestinal barrier function and alter mucosal immune responses (20). There are several possible microbial-related mechanisms implicated in ASD, such as dysbiosis-induced breakdown of gut integrity (21, 22), production of toxins (23), and immunological (24) and metabolic (23) abnormalities. A microbial shift within the gut of mice yields changes in serum metabolites and induces an autistic behavioral phenotype (25). Additionally, many studies have reported dysbiosis of the gut microbiota in individuals with ASD (26–28). However, different researchers reported various results. For example, Kang et al. reported a higher percentage of *Bacteroides* in the total detected microflora in children with ASD, whereas Strati et al. demonstrated a lower percentage of *Bacteroides* in children with ASD compared to controls (29, 30). Due to the currently available conflicting data, there is a need for a further investigation of the association between the gut microbiota and ASD. To better understand the effect of gut microbes on ASD, we carried out a meta-analysis to assess the differences in microbial populations between patients with ASD and age-matched controls. Such information is useful to design novel therapeutic strategies for modulating gut microbial populations in patients with ASD.

## Materials and Methods

### Search Strategy and Inclusion Criteria

We performed a systematic literature search of PubMed, Web of Science, and Cochrane databases up to July 2017 using the following terms: “autism (autism spectrum disorder) and microbiota” or “microbiome” or “dysbiosis” according to the Preferred Reporting Items for Systematic Reviews and Meta-Analyses (PRISMA) guidelines (31). The abstracts identified in this search were screened to eliminate clearly irrelevant studies. The criteria for study inclusion were as follows: 1) observational prospective and retrospective studies, case–control studies, or cohort studies; 2) investigating gut bacteria in children diagnosed with autism or ASD; 3) including information about sample size and prevalence of the specific bacteria assessed; and 4) written in English. Studies about non-human subjects as well as reviews, case reports, and duplicate publications were excluded. All articles providing sufficient information about the relationship between the gut microbiota and ASD were included.

The outcome of interest was the association between ASD and the gut microbiota. The definition of ASD was based on a physician’s diagnosis according to the *International Statistical Classification of Diseases and Related Health Problems, Tenth Revision* or a history of ASD reported by the parents of the children. Assessments of the biodiversity and composition of microbiota were based on stool sample testing using culture-dependent methods (32, 33), real-time polymerase chain reaction (PCR) (34), fluorescence *in situ* hybridization (FISH) (35), and pyrosequencing for bacterial 16S ribosomal ribonucleic acid (rRNA) genes (23, 29, 30, 36, 37). To conduct the meta-analyses, at least three studies were used to assess the bacteria. To maintain consistency within the present meta-analysis, all bacterial information were reviewed and selected before the final analyses, including bacterial taxonomy, percentage, and relative abundance. In general, gut bacteria were classified at different taxonomic levels from phylum (high taxonomic level) to genus (low taxonomic level). For consistency, the included studies were analyzed at the genus level. We contacted the investigators of the eligible studies if we were unable to extract data on bacterial abundance from the published articles.

### Quality Assessment

Four investigators independently carried out data extraction of the following items: author(s), publication year, study design, country, study population age, diagnosis of ASD, and effect size. Two reviewers completed the quality assessment independently. A set of structured criteria modified from previous studies was used to complete the quality assessment of publications. The total score ranged from 5 to 9 (with 9 as the highest), with a higher score indicating higher quality. In case of disagreement regarding the extracted data, discrepancies were resolved by consensus discussion.

### Statistical Analysis

A fixed-effects model and a random-effects model were used to report the most conservative result. Statistical heterogeneity was assessed using the *I* ^2^ value, which represents the percentage of total variation across different studies, owing to heterogeneity rather than chance. *I* ^2^ values of 25%, 50%, and 75% were related to low, moderate, and high heterogeneity (38). A random-effects model was applied when there was notable heterogeneity (*I* ^2^ index ≥ 50%); otherwise, a fixed-effects model was used. The standardized mean difference (SMD) measure of effect was used for the continuous variables (39). SMD > 0 indicates that participants with ASD have a higher bacterial abundance than controls, and SMD < 0 indicates participants with ASD have a lower bacterial abundance than controls. We also planned to analyze the influence of bias control in subgroup analyses as well as the evidence of publication bias and other small study effects using funnel plots and regression analyses. However, because of the limited number of studies, we only conducted subgroup analyses of studies that included participants with ASD or typically developing children. In our primary analysis, we included all published studies. The ratio of the bacterial percentage between children with ASD and controls was calculated to assess the relative abundance of bacteria in children with ASD compared to controls. All statistical analyses were carried out using Stata version 12.0 (Stata Corp, College Station, TX, USA).

## Results

### Characteristics of Included Studies

**Figure 1:**
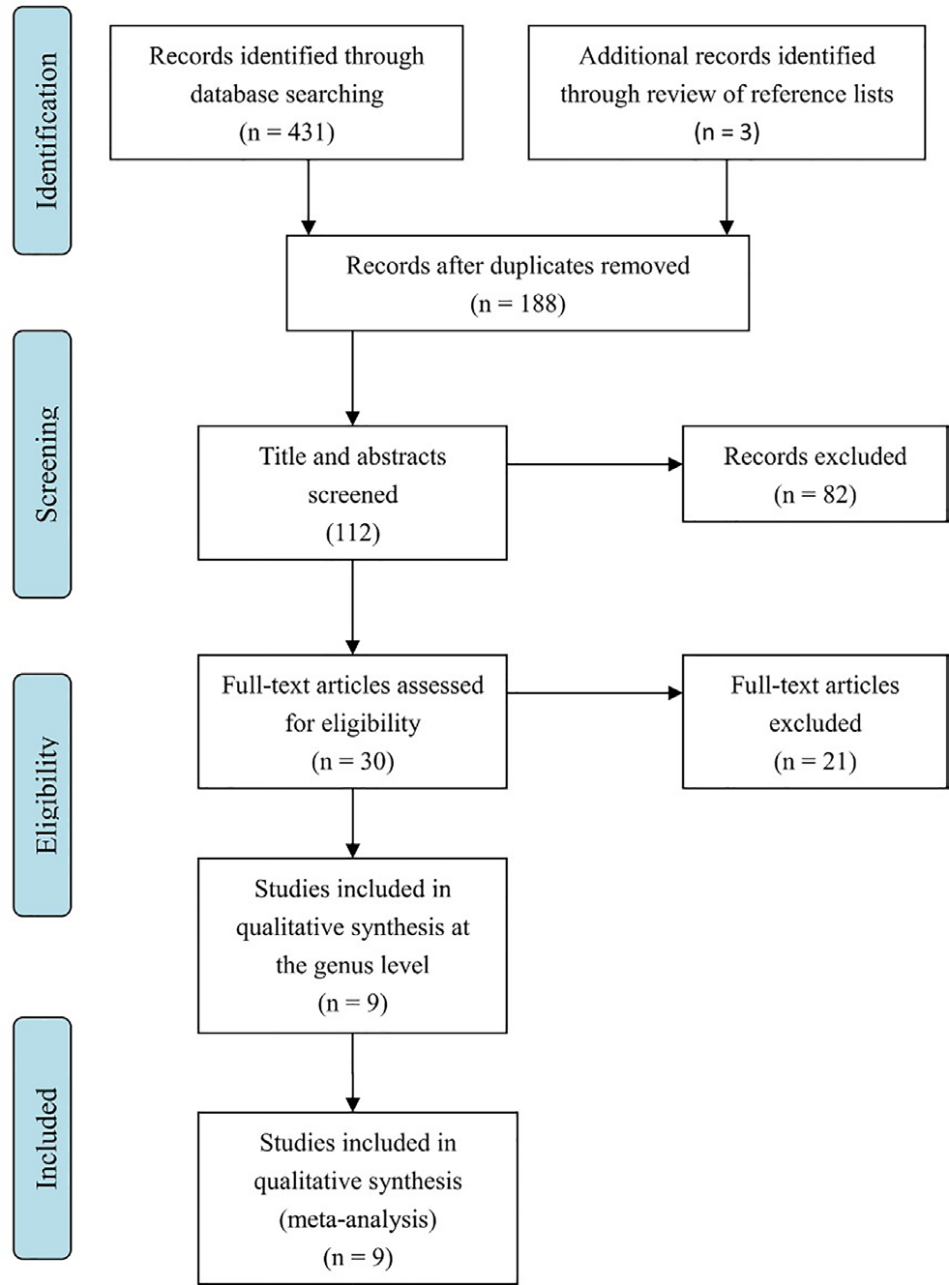
Flow diagram of study selection: article search strategy results.

Literature searches revealed 431 potentially eligible records (**Figure 1**). Three additional records were identified through a review of reference lists. After the review of the titles and abstracts and the removal of 246 duplicates, 112 publications were selected for a further review of the full texts. After the exclusion of records that were clearly irrelevant, involved nonhuman subjects, or have incomplete data, 30 full-text records were reviewed individually. Of these 30 articles, 9 studies were included in the present meta-analysis, as the remaining studies did not provide quantitative data about bacterial abundance or percentage in the report or after our request for essential details. In total, there were 254 participants with ASD and 167 age-matched typically developing controls with an age range from 6 to 11 years (**Table 1**). The diagnostic methods of ASD and comorbidity disease in the included studies are shown in **Table 2**. Gut microbiology was assessed using quantitative PCR (QPCR) or PCR, pyrosequencing, culture methods, or FISH (**Table 3**). Each study provided different types of bacteria for the meta-analysis (**Table 3**). The percentage and relative abundance of bacterial genera in the gut microbiota were used in the present analysis to avoid potential variation caused by different detection methods of the microbiota in the included papers. The absolute number of bacterial populations reported only in three studies was insufficient to perform a meta-analysis. The standard deviation of the mean was calculated for one study that only provided the mean and range (33) using a previously published method (40).

**Table 1:** Characteristics of the studies included in the analysis.

Author	Country	Study design	Autism (*n*)	Age (years)	Control (*n*)	Age (years)	Score
Parracho (35)	UK	NA	58	7 ± 3.76	10	6 ± 2.88	8
Kang (29)	USA	NA	20	6.7 ± 2.7	20	8.3 ± 4.4	7
Finegold (36)	USA	NA	11	2–13	8	2–13	7
Inoue (37)	Japan	NA	6	3–5	6	3–5	6
Strati (30)	Italy	Cohort	40	10 (5–17)	40	7 (3.6–12)	8
Wang (34)	Australia	NA	23	10.25 ± 0.75	9	9.5 ± 1.25	6
De Angelis (23)	Italy	NA	10	4–10	10	4–10	5
Adams (32)	USA	NA	58	6.91 ± 3.4	39	7.7 ± 4.4	7
Gondalia (33)	Australia	NA	28	2–14	25	NA	6

NA, not available.

**Table 2:** Diagnosis of autism and comorbidity disease in the included studies.

Author	Diagnosis of autism	Comorbidity	Assessment
Parracho (35)	ASD	GI disorder (91.4%)	Questionnaire
Kang (29)	ASD (Autism Diagnostics Interview)	GI problem	Questionnaire
Finegold (36)	Severe autism	GI symptoms (primarily constipation)	Pediatrician
Inoue (37)	By DSM-5, PARS, and M-CHAT	No GI disorder	NA
Strati (30)	By *Diagnostic and Statistical Manual of Mental Disorders, 5th Edition*	Constipation (12.5%)	NA
Wang (34)	ASD	Functional GI disorder	Questionnaire
De Angelis (23)	By DSM-IV-TR criteria	No	NA
Adams (32)	By a psychiatrist or a similar professional	Gut symptoms	Questionnaire
Gondalia (33)	ASD	NA	NA

DSM-5, Diagnostic and Statistical Manual of Mental Disorders, Fifth Edition; GI, gastrointestinal; ASD, autistic spectrum disorder; PARS, the Pervasive Developmental Disorders Autism Society Japan Rating Scale; M-CHAT, Modified Checklist for Autism in Toddlers; DSM-IV-TR, The Diagnostic and Statistical Manual of Mental Disorders, Fourth Edition; NA, not available.

**Table 3:** Assessment of the microflora in the included studies.

Author	Bacteria included in our analyses	Sample	Unit	Microbiology assessment
Parracho (35)	*Bacteroides*, *Bifidobacterium*, *Lactobacillus*	Fecal	Relative abundance	FISH (Cy3-labeled 16S rRNA probes)
Kang (29)	*Akkermansia*, *Bacteroides*, *Bifidobacterium*, *Faecalibacterium*, *Parabacteroides*, *Ruminococcus*	Fecal	Percentage	Pyrosequencing
Finegold (36)	*Akkermansia*, *Bacteroides*, *Clostridium*, *Faecalibacterium*, *Parabacteroides*, *Ruminococcus*	Fecal	Percentage	Pyrosequencing
Inoue (37)	*Akkermansia*, *Bacteroides*, *Bifidobacterium*, *Clostridium*, *Faecalibacterium*, *Ruminococcus*, *Parabacteroides*	Fecal	Percentage	Pyrosequencing
Strati (30)	*Akkermansia*, *Bacteroides*, *Bifidobacterium*, *Clostridium*, *Faecalibacterium*, *Ruminococcus*, *Parabacteroides*	Fecal	Percentage	Pyrosequencing
Wang (34)	*Bacteroides*, *Bifidobacterium*, *E. coli*, *Enterococcus*, *Lactobacillus*	Fecal	Relative abundance	QPCR (various bacterial primers)
De Angelis (23)	*Akkermansia*, *Bacteroides*, *Bifidobacterium*, *Clostridium*, *Enterococcus*, *Faecalibacterium*, *Parabacteroides*, *Ruminococcus*	Fecal	Percentage, relative abundance	Pyrosequencing
Adams (32)	*Bifidobacterium*, *E. coli*, *Enterococcus*, *Lactobacillus*	Fecal	Relative abundance	Culture (colony-forming units)
Gondalia (33)	*E. coli*	Fecal	Relative abundance	Culture (colony-forming units)

FISH, fluorescence in situ hybridization; rRNA, ribosomal ribonucleic acid; QPCR, real-time quantitative PCR detecting system.

#### Akkermansia

**Figure 2:**
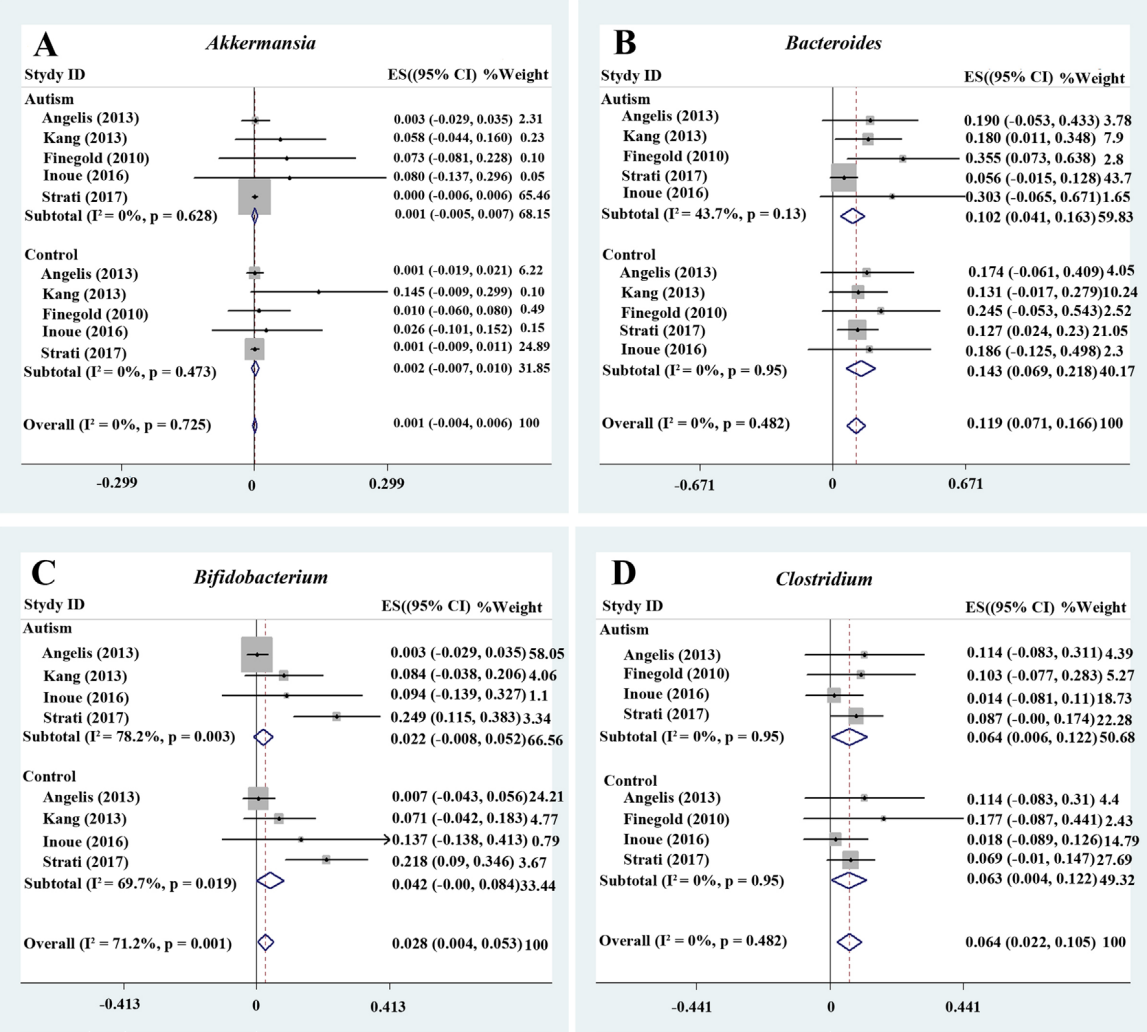
Forest plot of percentages of *Akkermansia*, *Bacteroides*, *Bifidobacterium*, and *Clostridium* in ASD. **(A–D)** Percentages of *Akkermansia*, *Bacteroides*, *Bifidobacterium*, and *Clostridium* in the total detected microflora, respectively. Fixed-effects models were used to assess *Akkermansia*, *Bacteroides*, and *Clostridium*. A random-effects model was used to analyze *Bifidobacterium*, contributing to higher between-study heterogeneity (*I* ^2^ > 50%). The pooled percentages of *Akkermansia*, *Bacteroides*, *Bifidobacterium*, and *Clostridium* from the included studies were 0.1%, 11.9%, 2.8%, and 6.4%, respectively.

We analyzed the percentage of *Akkermansia* from five trials (**Figure 2A**). A fixed-effects meta-analysis showed that the percentage of *Akkermansia* in the total detected microflora was 0.1% in participants with ASD [95% confidence interval (CI): −0.005 to 0.007] compared to 0.2% in typically developing children (95% CI: −0.007 to 0.01). There was no evidence of between-study heterogeneity (*I* ^2^ = 0%; **Figure 2A**). However, its effect size (*Z* = 0.44, *P* = 0.658) was relatively small. The ratio of bacterial percentage between the ASD group (0.1%) and the control group (0.2%) was 0.5. The percentage of *Akkermansia* in patients with ASD was clearly lower compared to controls (**Figure 5A**).

#### Bacteroides

**Figure 3:**
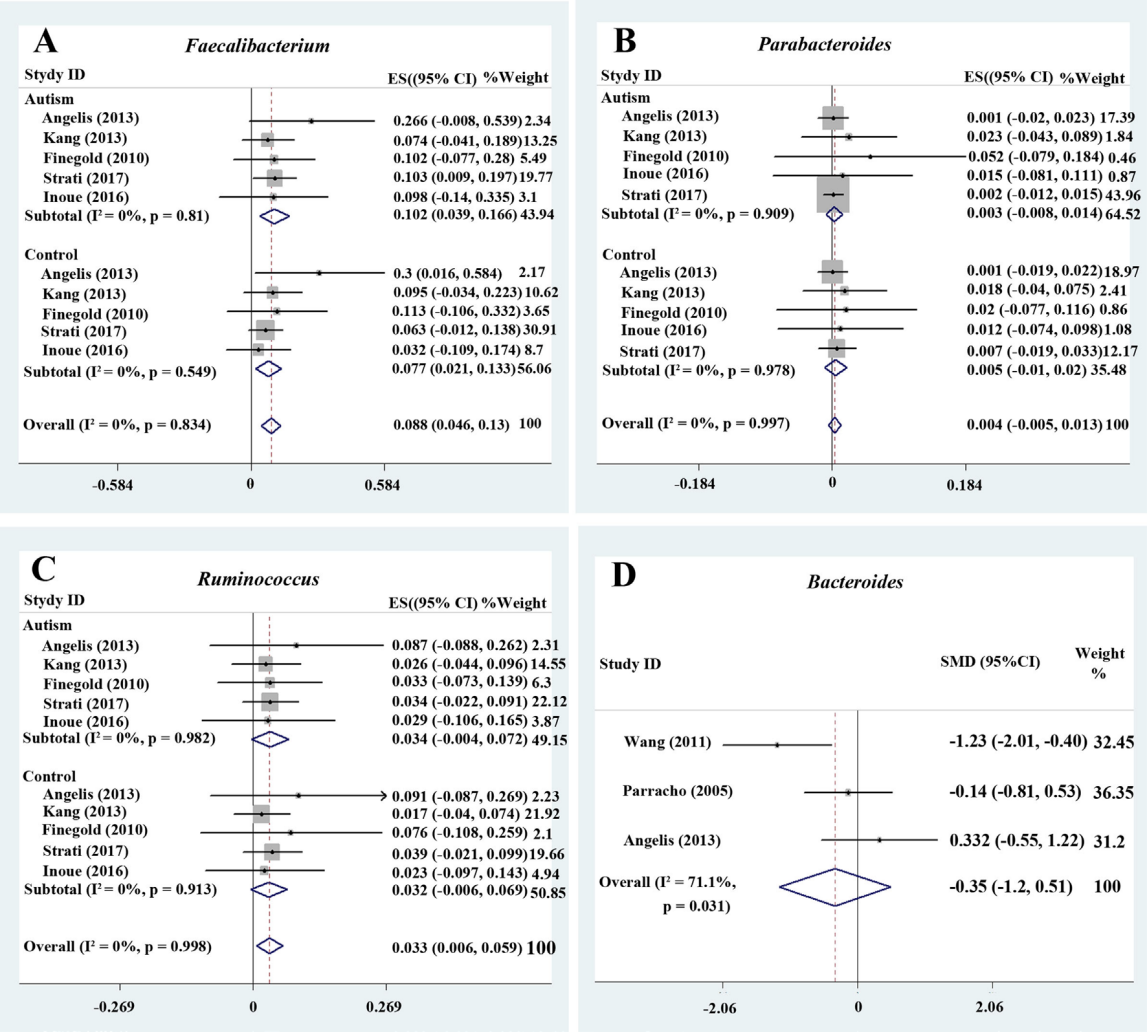
Forest plot of percentages of *Faecalibacterium*, *Parabacteroides*, *Ruminococcus*, and *Bacteroides* in autism spectrum disorder (ASD). **(A–C)** Percentages of *Faecalibacterium*, *Parabacteroides*, and *Ruminococcus* in the total detected microflora, respectively. Fixed-effects models were used to assess *Faecalibacterium*, *Parabacteroides*, and *Ruminococcus*. The pooled percentages of *Faecalibacterium*, *Parabacteroides*, and *Ruminococcus* from the included studies were 8.8%, 0.4%, and 3.3%, respectively. **(D)** Relative abundance of *Bacteroides*. A random-effects model was used to analyze *Bacteroides*, contributing to higher between-study heterogeneity (*I* ^2^ > 50%).

*Bacteroides* is a Gram-negative bacterium and is one of the earliest colonizing and most abundant constituents of the gut microbiota and may induce an anti-inflammatory milieu (41). A fixed-effects meta-analysis showed that the percentage of *Bacteroides* in the total detected microflora was 10.2% (95% CI: 0.041–0.163) in children with ASD but 14.3% in typically developing children (95% CI: 0.069–0.218). There was low between-study heterogeneity within the ASD group (*I* ^2^ = 43.7%; **Figure 2B**). The effect size (*Z* = 4.92, *P* = 0.000) was significant and large. The ratio of the bacterial percentage between the ASD group (10.2%) and the control group (14.3%) was 0.71 (**Figure 5A**). Furthermore, a random-effects model also showed a lower abundance of *Bacteroides* in participants with ASD compared to controls (SMD −0.35, 95% CI: −1.2 to 0.51; **Figure 3D**). However, its effect size (*Z* = 0.8, *P* = 0.427) was relatively small.

#### Bifidobacterium

**Figure 4:**
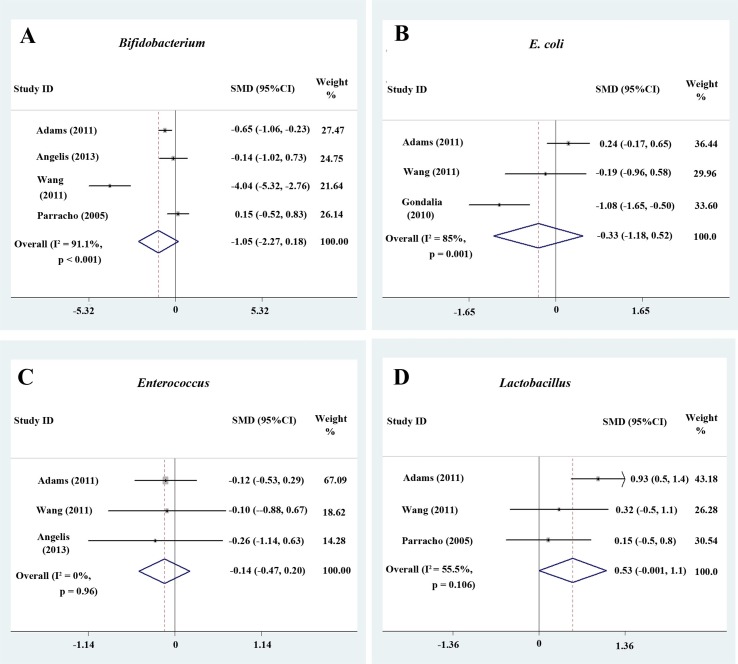
Forest plot of the relative abundance of *Bifidobacterium*,* E. coli*,* Enterococcus*, and *Lactobacillus* in ASD. **(A–D)** Relative abundance of *Bifidobacterium*,* E. coli*,* Enterococcus*, and *Lactobacillus*. Random-effects models were used to analyze *Bifidobacterium*,* E. coli*, and *Lactobacillus*, contributing to higher between-study heterogeneity (*I* ^2^ > 50%), except *Enterococcus*.

**Figure 5:**
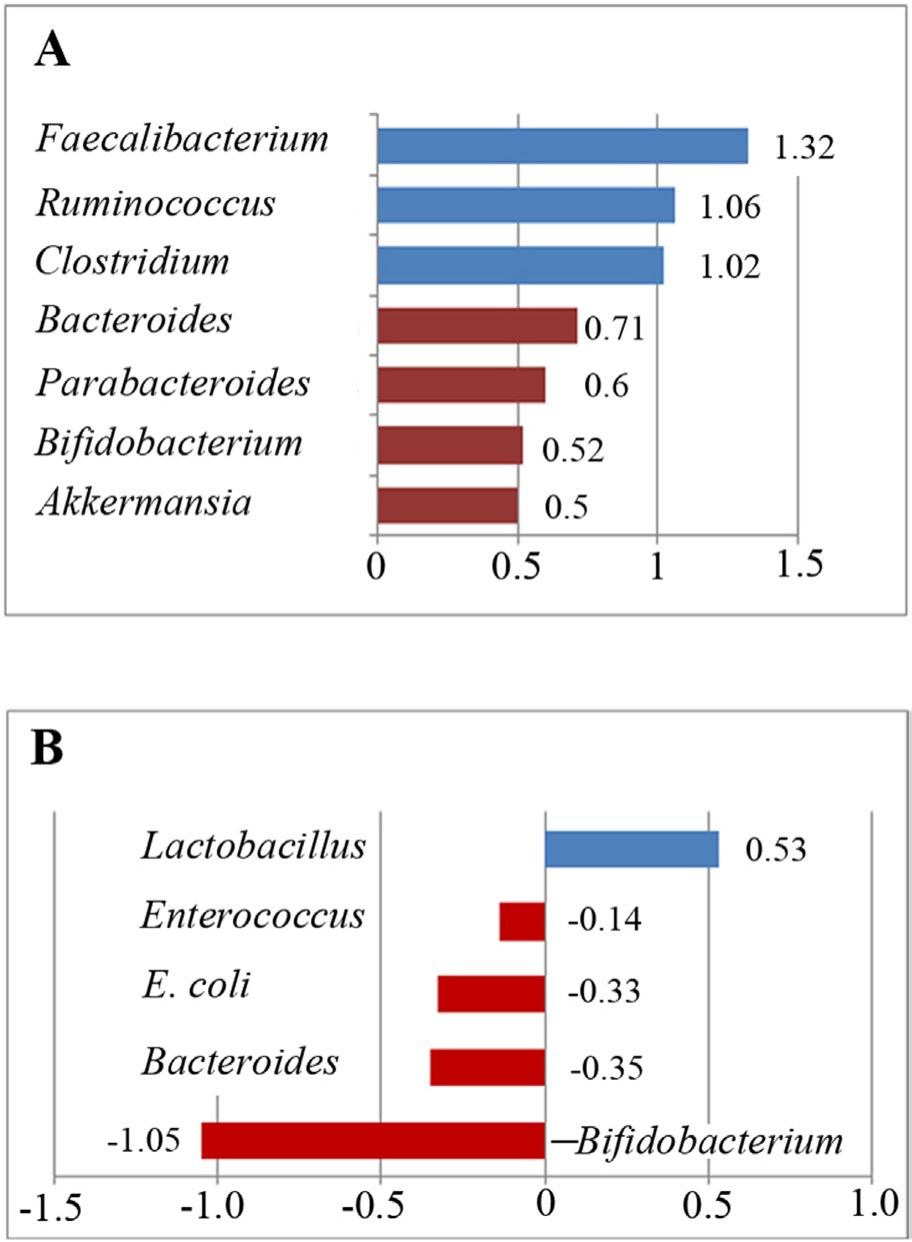
Relative abundance of the included bacteria in the meta-analysis. **(A)** Ratio of the bacterial percentages in children with ASD and typically developing children. A value greater than 1 indicates higher abundance in children with ASD (*Faecalibacterium*, *Ruminococcus*, and *Clostridium*), whereas a value less than 1 indicates lower abundance in children with ASD compared to controls. **(B)** Relative abundance of the gut microbiota in children with ASD. A positive value indicates higher abundance in children with ASD (*Lactobacillus*), whereas a negative value indicates lower abundance in children with ASD.

*Bifidobacterium* has long been used as a probiotic to alleviate various diseases by changing the gut microbiota composition (34, 42). A random-effects meta-analysis showed 2.2% of *Bifidobacterium* in the total detected microflora of children with ASD (95% CI: −0.008 to 0.052), whereas the percentage in typically developing children was 4.2% (95% CI: −0.00 to 0.084) with moderate between-study heterogeneity (*I* ^2^ = 78.2% and 69.7%, respectively; **Figure 2C**). The effect size (*Z* = 2.27, *P* = 0.023) was significant and moderate. The ratio of the bacterial percentage between the ASD group (2.2%) and the control group (4.2%) was 0.52 (**Figure 5A**). The percentage of *Bifidobacterium* in patients with ASD was clearly lower compared to controls. Furthermore, a random-effects model showed a lower abundance of *Bifidobacterium* in children with ASD (SMD −1.05, 95% CI: −2.27 to 0.18; **Figure 4A**).

#### Faecalibacterium

Five studies were used to evaluate the percentage of *Faecalibacterium* (**Figure 3A**). A fixed-effects meta-analysis showed that the percentage of *Faecalibacterium* in the total detected microflora of children with ASD was 10.2% (95% CI: 0.039–0.166), clearly higher than that of typically developing children (7.7%; 95% CI: 0.021–0.133). The effect size (*Z* = 4.12, *P* = 0.000) was significant and large. The ratio of the bacterial percentage between the ASD group (10.2%) and the control group (7.7%) was 1.32 (**Figure 5A**).

#### Ruminococcus

*Ruminococcus* is an anaerobic Gram-positive coccus that can be found in the GI tract (43, 44). The percentages of *Ruminococcus* in the total detected microflora were assessed. A fixed-effects meta-analysis showed 3.4% and 3.2% for children with ASD and typically developing controls, respectively (95% CI: −0.004 to 0.072 and −0.006 to 0.069, respectively). There was no evidence of between-study heterogeneity (*I* ^2^ = 0%; **Figure 3C**) with respect to *Ruminococcus* percentages. The effect size (*Z* = 2.42, *P* = 0.016) was significant and moderate. The ratio of the bacterial percentage between the ASD group (3.4%) and the control group (3.2%) was 1.06 (**Figure 5A**). The percentage of *Ruminococcus* in patients with ASD was slightly higher compared to controls.

#### Clostridium, Parabacteroides, Escherichia coli, Enterococcus, and Lactobacillus

A fixed-effects meta-analysis showed that the percentage of *Clostridium* in the total detected microflora of children with ASD was 6.4% (95% CI: 0.006–0.122), similar to that of typically developing children (6.3%; 95% CI: 0.004–0.122; **Figure 2D**). Additionally, the fixed-effects meta-analysis also showed that the percentage of *Parabacteroides* in the total detected microflora of children with ASD was 0.3% (95% CI: −0.008 to 0.014) compared to 0.5% in typically developing children (95% CI: −0.01 to 0.02; **Figure 3B**). The ratio of the bacterial percentage between the ASD group (0.3%) and the control group (0.5%) was 0.6, indicating a decreased percentage in children with ASD (**Figure 5A**).

The random-effects and fixed-effects models showed a lower relative abundance of *E. coli* and *Enterococcus* in children with ASD compared to controls (SMD −0.33, 95% CI: −1.18 to 0.52 and SMD −0.14, 95% CI: −0.47 to 0.20, respectively; **Figure 4B** and **4C**). A random-effects meta-analysis showed a higher relative abundance of *Lactobacillus* in children with ASD (SMD 0.53, 95% CI: −0.001 to 1.1; **Figure 4D**). The pooled relative abundance of *Lactobacillus*, *Enterococcus*, *E. coli*, *Bacteroides*, and *Bifidobacterium* in children with ASD are shown in **Figure 5B**.

## Discussion

### Alterations of the Gut Microbiota in Patients With ASD

The gut microbiota plays a major role in human physiology and pathology (45–47). Both experimental and clinical cross-sectional studies showed that patients with ASD had alterations of the gut microbiota (48). These alterations were potentially relevant to behavioral and GI symptoms that are correlated with the severity of ASD (7, 43, 49–52), suggesting that the gut-brain axis participates in the pathogenesis of ASD (18, 53, 54).

Although several reviews suggested a microbiota alteration in patients with ASD (28, 30, 55–60), this is the first meta-analysis that systematically reviewed published data and examined microbiota alterations in patients with ASD. Standardized data collection, strict inclusion criteria, and multiple statistical tools were used to ensure the most accurate assessment. The present meta-analysis found that neither there were significant changes in the intestinal microbial diversity nor single microbial species may be perceived as “ASD-promoting microbes.”Our analyses showed that participants with ASD had a lower abundance of *Akkermansia, Bacteroides*, *Bifidobacterium*, *E. coli*, and *Enterococcus*, a higher abundance of *Faecalibacterium* and *Lactobacillus*, and a slightly increased abundance of *Ruminococcus* and* Clostridium*. It is possible that the reduced levels of beneficial bacteria combined with the increased levels of harmful bacteria contribute together to ASD symptoms. Our analysis is consistent with previous reviews (61), with one exception of *Clostridium*. Several studies showed there was a higher level of *Clostridium* in individuals with ASD compared to controls and hypothesized that *Clostridium* can produce neurotoxins and contribute to ASD (62, 63). The current analysis showed slightly increased levels of *Clostridium* and *Ruminococcus*, indicating that further studies should be performed to confirm these trends. In contrast, there is potentially a decrease in “beneficial” bacteria in patients with ASD (34, 64). This notion is further supported by a recent study showing that the supplementation of *Bifidobacterium* species-containing probiotics improves specific ASD symptoms (65).

### Potential Mechanisms of the Gut Microbiota in ASD

The role of the gut microbiota in development and disease is not yet well understood. Potential mechanisms by which microbiota impacts the gut-brain axis and ASD progression involve inflammatory and metabolic pathways and alteration of epithelial barrier integrity. First, the abundance of *Faecalibacterium* may play a role in systemic immunity dysfunction. The abundance of *Faecalibacterium* was significantly higher in children with ASD compared to controls (30). The expression levels of interferon response factors 7 and 9 showed a strong correlation with the abundance of *Faecalibacterium* in fecal microbiota, which could produce substances that activate type I interferon signaling (66). In contrast, protective bacteria such as *Bifidobacterium* were decreased in abundance in individuals with ASD across the analyzed studies. Bifidobacteria are major producers of lactic acid, which suppress the growth of pathogens such as *E. coli* across the epithelium, reduce inflammation in the gut, and cooperate with the immune system (67, 68). In the present study, lower levels of *Bifidobacterium* and higher levels of *Lactobacillus* suggested an imbalance in beneficial bacteria. Decreased levels of *Bifidobacterium* and metabolites of free amino acids and short-chain fatty acids (SCFA) in the feces may also contribute to the development of ASD (23). A low level of SCFA was possibly related to probiotic usage, lower saccharolytic fermentation by beneficial bacteria, or increased gut permeability, subsequently exacerbating autistic symptoms (32). *Akkermansia* is a mucin-degrading bacterium present in the gut of typically developing adults. A lower abundance of *Akkermansia* in children with ASD could indicate a thinner GI mucus barrier in children with ASD compared to controls; the result might reflect an indirect evidence of impaired gut permeability in children with ASD (34). Second, animal studies have shown effects of the gut microbiota on neurodevelopment, suggesting that intestinally derived lipopolysaccharides can increase anxiety-like behavior in mice (69–71). Furthermore, gut microbial populations in ASD may produce toxic products, including neurotoxins that influence distal sites such as the brain, and exert systemic effects (35). Third, microbiota and their metabolites are essential in maintaining both white matter and epithelial barrier integrity, which is important for normal brain development and function (72). The development of the blood-brain barrier is now well established to be contingent upon the presence of commensal gut flora (10, 11). Additionally, diet-specific gut microbiota populations potentially influence white matter integrity in rats (59). These studies reveal a potential mechanism for the gut microbiota in influencing the brain-gut-enteric microbiota axis and contribute to the understanding of the role of the brain-gut axis in the pathogenesis of ASD.

Children with ASD also have a high rate of GI symptoms, which correlate with ASD severity (32, 73) and are associated with ASD-relevant emotional and behavioral problems (74, 75). More than 50% of GI symptoms may be due to dysbiotic gut microbiota, including increased *Ruminococcaceae* (76, 77). In our meta-analysis, only two studies enrolled children with ASD who had no GI symptoms (23, 37), and one study did not provide details about GI symptoms (33). Collectively, the studies included in the current analysis, however, indicate a high incidence of GI symptoms in children with ASD. The GI symptoms might be related to the ubiquity of food selectivity in this population, as the dietary patterns often associated with ASD involve a high intake of processed food and lack fiber-containing fruits and vegetables. Gastroesophageal reflux, gastroenteritis, food allergies, and inflammatory bowel disease are also more common in children with ASD, probably contributing to the development of GI symptoms (78).

### Limitations of the Study

The meta-analysis is inherently limited by the included studies. First, the study design, specificity, and sensitivity of the detection methods used in the included studies varied. The studies included in our analysis mainly used culture-based methods, PCR and pyrosequencing, to analyze the changes of particular bacterial groups, which might underestimate the complexity of the gut microbiota. Indeed, we found that suitable analytical and statistical methods are critical to detect the alterations of the abundance of some gut microbiota in patients with ASD. Second, many reports had relatively small sample sizes with only two of the nine studies recruiting more than 50 participants with ASD. Significant heterogeneity was found between studies when the data were pooled. Finally, our study only analyzed bacterial percentages and abundance at the genus level due to the insufficiency of data for various bacterial taxonomies. Further broad-based, longitudinal, unbiased studies of fecal microbial populations in patients with ASD and age-matched controls using next-generation sequencing will be more informative for clarifying ASD-associated dysbiosis.

## Conclusion

Our review summarized the association between ASD and gut microbiota composition. Participants with ASD had a lower abundance of *Akkermansia, Bacteroides*, *Bifidobacterium*, *E. coli*, and *Enterococcus*, a higher abundance of *Faecalibacterium* and *Lactobacillus*, and a slightly increased abundance of *Ruminococcus* and *Clostridium*. There were important differences, such as the abundance of *Akkermansia*, *Bifidobacterium*, *Bacteroides*, *E. coli*, and *Lactobacillus* between the microbiota of children with ASD and typically developing children. Our analysis warrants additional prospective cohort studies to evaluate the influence of the microbiota in the pathogenesis of ASD and associated GI symptoms. A future impact of such studies could potentially guide the implementation of dietary/probiotic interventions impacting the gut microbiota in patients with ASD.

## Author Contributions

FL and JL designed and supervised the study. MX and XX performed the data analysis and interpretation and wrote the manuscript. All authors read and approved the final version to be published and agreed to be accountable for all aspects of the work.

## Funding

This study was supported by grants from the National Natural Science Foundation of China (no. 81571031 and 8171101223), the Shanghai Committee of Science and Technology (no. 17XD1403200), the Shanghai Municipal Education Commission (Research Physician Project; no. 20152234), the Shanghai Municipal Commission of Health and Family Planning (no. GDEK201709, 2017ZZ02026, and 2017EKHWYX-02), the Shanghai Shenkang Hospital Development Center (no. 16CR2025B), and the Shanghai Municipal Health Commission (no. 2019SY068).

## Conflict of Interest Statement

The authors declare that the research was conducted in the absence of any commercial or financial relationships that could be construed as a potential conflict of interest.
